# Potential hot spot for *de novo* mutations in *PTCH1* gene in Gorlin syndrome patients: a case report of twins from Croatia

**DOI:** 10.3325/cmj.2018.59.20

**Published:** 2018-02

**Authors:** Vesna Musani, Petar Ozretić, Diana Trnski, Maja Sabol, Sanja Poduje, Mateja Tošić, Mirna Šitum, Sonja Levanat

**Affiliations:** 1 Laboratory for Hereditary Cancer, Ruđer Bošković Institute, Zagreb, Croatia; 2 University Department of Dermatovenereology, University Hospital “Sestre milosrdnice”, Zagreb, Croatia; 3 Clinic of Dermatovenereology Sniježana Nakić Tošić, Makarska, Croatia

## Abstract

We describe a case of twins with sporadic Gorlin syndrome. Both twins had common Gorlin syndrome features including calcification of the falx cerebri, multiple jaw keratocysts, and multiple basal cell carcinomas, but with different expressivity. One brother also had benign testicular mesothelioma. We propose this tumor type as a possible new feature of Gorlin syndrome. Gorlin syndrome is a rare autosomal dominant disorder characterized by both developmental abnormalities and cancer predisposition, with variable expression of various developmental abnormalities and different types of tumors. The syndrome is primarily caused by mutations in the Patched 1 (*PTCH1*) gene, although rare mutations of Patched 2 (*PTCH2)* or Suppressor of Fused (*SUFU*) genes have also been found. Neither founder mutations nor hot spot locations have been described for *PTCH1* in Gorlin syndrome patients. Although *de novo* mutations of the *PTCH1* gene occur in almost 50% of Gorlin syndrome cases, there are a few recurrent mutations. Our twin patients were carriers of a *de novo* mutation in the *PTCH1* gene, c.3364_3365delAT (p.Met1122ValfsX22). This is, to our knowledge, the first Gorlin syndrome-causing mutation that has been reported four independent times in distant geographical locations. Therefore, we propose the location of the described mutation as a potential hot spot for mutations in *PTCH1*.

Gorlin syndrome, or nevoid basal cell carcinoma syndrome (NBCCS), is a rare autosomal dominant disorder characterized by developmental abnormalities including cleft palate and skeletal abnormalities, such as bifid ribs, palmar and plantar pits, macrocephaly, and calcification of the falx cerebri, and a predisposition to the development of various tumors, such as basal cell carcinomas (BCCs), desmoplastic medulloblastomas, jaw cysts, and fibromas of the ovaries and heart, which occur at different ages (1). Although this disorder has almost complete penetrance, it is sometimes difficult to diagnose due to its highly variable features both within and outside families and very low estimated prevalence of only 1 in 57 000 to 1 in 256 000 persons (2,3).

This complex disorder is mostly caused by mutations of the Patched 1 (*PTCH1*) gene, although rare mutations of Patched 2 (*PTCH2)* or Suppressor of Fused (*SUFU*) genes have also been found (2). They are all members of the Hedgehog-Gli (Hh-Gli) signaling pathway, which is an important developmental pathway involved in body symmetry patterning and organ differentiation (2,4). It is also involved in patterning of steroidogenic tissues and sex determination during embryonic development (5). Hh-Gli signaling pathway, which is normally active only during embryonic development and in stem cells, is inactive in adult differentiated tissues (6), becomes dysregulated and overly active in many different cancer types (7).

This report describes twins with sporadic Gorlin syndrome, with possibly the first truly recurrent *PTCH1* mutation. One of the twins also had a rare tumor that might be a new Gorlin syndrome feature.

## CASE REPORT

We present a case of monozygotic twins, born in 1981, with Gorlin syndrome. At age 11, the first twin presented with urinary tract infection with hematuria. During the examination, several pigmented moles on the patients’ skin, winged scapula (scapula alata), kyphosis, and funnel chest were noted. Relevant family history was taken, but the Gorlin syndrome was not suspected (Table 1). At age 13, he developed benign paratesticular mesothelioma. At age 17, when he developed the first multiple jaw cysts and only after his brother already been surgically treated for his jaw cyst at the same clinic, the Gorlin syndrome was suspected for both brothers. Eventually, they were diagnosed as sporadic cases.

**Table 1:** Medical history timeline of both brothers. G1 – first patient, G2 – second patient.

Year	G1		G2	
	Summary of initial and follow-up visits	Diagnostic/interventions	Summary of initial and follow-up visits	Diagnostic/interventions
1992 (aged 11)	Urinary tract infection with hematuria	Treated with antibiotics; Several nevi detected on the skin		
1994 (aged 13)	Benign paratesticular mesothelioma	Surgical removal		
1997 (aged 16)			Single jaw cyst	Surgical removal
1998 (aged 17)	Multiple jaw cysts	Surgical removal, sporadic Gorlin syndrome suspected	Multiple jaw cysts	Surgical removal, sporadic Gorlin syndrome suspected
2013 (aged 32)	Multiple basal cell carcinomas (BCC)	Surgical excision of larger BCCs, cryotherapy for smaller, Gorlin syndrome diagnosed, Patched 1 gene (*PTCH1*) testing indicated	Multiple basal cell carcinomas	Surgical excision of larger BCCs, cryotherapy for smaller, Gorlin syndrome diagnosed, *PTCH1* testing indicated
2013 (aged 32)	*PTCH1* mutation testing	*PTCH1* mutation found, Gorlin syndrome confirmed	*PTCH1* mutation testing	*PTCH1* mutation found, Gorlin syndrome confirmed

Thorough medical exams were conducted and detailed family history was taken. The patient had calcification of the falx cerebri, bridging of sella turcica, hypoplasia of frontal parts of the ribs VI and VII on the right front side, anterior open bite, partial hypoplasia of tooth enamel, multiple jaw keratocysts, and numerous nevoid skin changes.

The second brother had his first jaw cyst diagnosed at the age of 16, and multiple jaw cysts at the age of 17. He also had a slight calcification of the falx cerebri, calcification along the intraparietal suture and tabula interna of the frontal bone, deformity of left hemithorax, and several nevoid skin changes.

Their mother had a thyroid tumor, liver and kidney cysts, and hypertension. Their father had several jaw cysts diagnosed several years before. One grandfather had jaw cysts later in life (8). Other family members showed no relevant clinical features.

In 2013, at age 32, after both brothers developed multiple basal cell carcinomas, Gorlin syndrome diagnosis was confirmed and additional tests were performed. The brain scan of the first brother showed cerebral lateral ventricular asymmetry, asymmetry of temporal horns, slight frontal cortical atrophy, and the radiographic images of the skeleton showed discrete thinning of the bone structure in the left femur, slight calcification of the right acetabulum, mild hypertrophy of the right femoral head-neck junction, and thinning of bone structure in the right thumb.

The second twin’s brain scan showed slight frontal cortical atrophy and the radiographic images of the skeleton described discrete thinning of the bone structure in the distal third of both femurs and several bones of the right hand.

At that time, testing for *PTCH1* gene mutations was recommended. The blood samples from both brothers, their parents, and their unaffected sister were obtained after signing informed consent forms. The study was conducted according to the Declaration of Helsinki principles.

**Figure 1:**
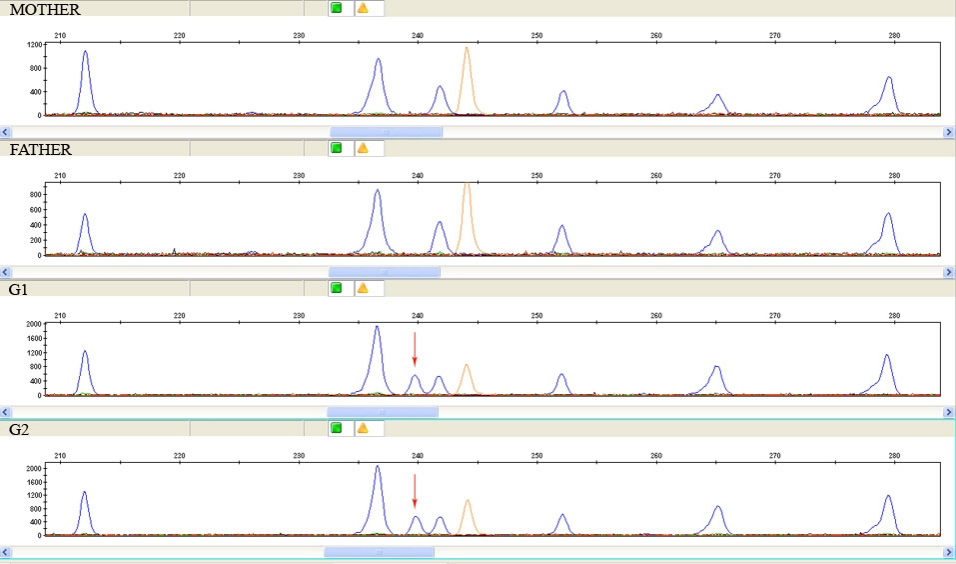
Quantitative multiplex polymerase chain reaction (PCR) analysis showing the deletion in exon 20 of Patched 1 gene (*PTCH1*) seen as an extra peak in brothers (G1 and G2), whereas in the parents the extra peak is absent.

Quantitative multiplex polymerase chain reaction (PCR) was used for the analysis of large rearrangements (9) and high-resolution melting analysis for point mutations (10), followed by sequencing in both directions. Quantitative multiplex PCR discovered a small deletion in exon 20 not present in either parent (Figure 1). Sequencing showed a *PTCH1* mutation c.3364_3365delAT (p.Met1122ValfsX22), predicted to occur in the transmembrane domain 11. Since the mutation was absent in the parents, we wanted to determine whether the mutation occurred on the maternal or paternal allele. All 14 known polymorphisms previously found in Croatia were typed in both parents (Table 2). Only three polymorphisms were found. One was not informative, and while both c.1504-51C>G in intron 10 and c.1647C>T in exon 12 differed in parents and sons, with more than 20 000 bp away from the mutation site, it was not possible to determine the strand of origin. The c.1647C>T polymorphism seems to be a very rare Croatian polymorphism, previously found in only a few samples (10).

**Table 2:** Patched 1 gene (*PTCH1*) polymorphisms typed for linkage analysis in twins with Gorlin syndrome and their parents*

Polymorphism	rs #	F	M	G1	G2
c.-4_-3insGGC	rs587780530	WT	WT	WT	WT
c.202-538delC	rs11362678	WT	WT	WT	WT
c.318C>T	rs1805153	WT	WT	WT	WT
c.735A>G	rs1805154	WT	WT	WT	WT
c.747-55T>C	rs2297087	WT	WT	WT	WT
c.1504-51C>G	rs574688	HO	HET	HET	HET
c.1504-8T>C	rs2277184	WT	WT	WT	WT
c.1641C>T	rs2066830	WT	WT	WT	WT
c.1647C>T	ss2137510529	WT	HET	HET	HET
c.1665T>C	rs1805155	WT	WT	WT	WT
c.1686C>T	rs2066836	WT	WT	WT	WT
c.2560 + 9G>C	rs2066829	WT	WT	WT	WT
c.3141T>G	rs2066835	WT	WT	WT	WT
c.3944C>T	rs357564	HET	HET	HO	HO

*Abbreviations: rs # – a reference single nucleotide polymorphism identification (SNP ID) number, ss # – a submitted SNP ID number, M – mother, F – father, G1 – first patient, G2 – second patient, WT – wild type, HET – heterozygote, HO – rare homozygote.

Since 2013, both brothers have been under regular observations at the Dermatologic Surgery of the University Department of Dermatovenereology, University Hospital “Sestre milosrdnice” and have had multiple basal cell carcinomas removed on more than 20 occasions.

## DISCUSSION

This report describes a case of twins with sporadic Gorlin syndrome. The patients are carriers of a *de novo* mutation in the *PTCH1* gene, c.3364_3365delAT (p.Met1122ValfsX22). Both have common Gorlin syndrome features, but one brother also had benign testicular mesothelioma. We propose that this tumor type might be a possible new Gorlin syndrome feature.

Gorlin syndrome is a complex disease and rarely occurring symptoms can easily be overlooked, especially during childhood, as was the case with the brothers’ first jaw cysts and skin nevi. The first brother has a more severe phenotype, even though they are monozygotic twins. This has been noted before for monozygotic twins affected by Gorlin syndrome, and has mostly been attributed to environmental factors (11).

The first brother developed benign paratesticular mesothelioma, which is not an established Gorlin syndrome feature, but it might be of some interest. These tumors are rare, benign, and typically occur in men in their twenties to sixties (12). Malignant mesotheliomas can have mutations in *PTCH1*, *Smoothened* (*SMO*), and *SUFU* genes (13). Mesotheliomas in both their benign and malignant forms are derived from the mesenchymal tissue, most often during the development and differentiation of genital tract organs (14). Since the Hh-Gli pathway is involved in patterning of steroidogenic tissues, it is not surprising to find this type of tumor in the Gorlin syndrome patient. Benign mesothelioma has likely not been associated with the syndrome before because it is rare and usually detected only incidentally.

The brothers were diagnosed as sporadic cases of Gorlin syndrome, because their parents do not have the constitutive *PTCH1* mutation. It is possible that the father has a mosaic mutation, since he has some features of Gorlin syndrome, but not the mutation in his constitutional DNA. Unfortunately, there was no tumor material available to test. *De novo PTCH1* mutations are quite common, in up to 50% of patients, and the same mutation is rarely found twice (3). Interestingly, the mutation we describe here has been reported three times so far, in Australia and New Zealand (15), USA (3) and Japan (16). As in our patients, all cases were sporadic (personal communication for the USA and Japan patients). It is likely that the structure and the sequence of DNA at this position is a fragile site prone to damage and is more likely to be affected by random DNA damaging events.

Although founder *PTCH1* mutations have not been described in the literature, several recurrent mutations have been reported (17). Until this report, a recurrent mutation has been reported only three times and only in family cases. Sporadic *de novo* mutations were only reported twice for each mutation.

This is, to our knowledge, the first mutation that is described four times in very distant geographical locations, and at least three times as a sporadic mutation. Therefore, we would like to propose the c.3364_3365delAT mutation as a potential hot spot for mutations in *PTCH1*.
